# Post-Radiotherapy Complications in Ewing Sarcoma: A Case Report and Literature Review

**DOI:** 10.7759/cureus.51579

**Published:** 2024-01-03

**Authors:** Zubir S Rentiya, Resheek Nerella, Ishaan Thassu, Pugazhendi Inban, Ihab Sheikh Hanafi, Parvinder Kaur

**Affiliations:** 1 Department of Radiation Oncology & Radiology, University of Virginia School of Medicine, Charlottesville, USA; 2 Trauma Surgery, University of Illinois College of Medicine Peoria (UICOMP), Illinois, USA; 3 Internal Medicine, Calcutta National Medical College, Kolkata, IND; 4 Department of General Medicine, Government Medical College, Omandurar, Chennai, IND; 5 Internal Medicine, Spartan Health Sciences University, Vieux Fort, LCA; 6 Internal Medicine, Crimean State Medical University, Simferopol, UKR

**Keywords:** chemotherapy, metastatic es, radiation therapy, immunohistochemistry, ewing's sarcoma (es)

## Abstract

Ewing's sarcoma (ES), the second most prevalent malignant osseous tumor in children and adolescents, primarily affects the extremities' long bones and pelvic region. Characterized by its aggressive growth, ES often presents with symptoms like swelling, pain, and neurological deficits, impacting various skeletal sites. ES involving the spine, particularly the sacral region, poses a significant challenge due to its rarity, aggressive nature, and limited sensitivity to treatments. We report the case of an 18-year-old male with recurrent metastatic ES presenting with fever, cough, and a lesion in the right humerus. Despite prior treatments and complications including spinal metastasis and cord compression, the patient's condition deteriorated, resulting in an unfortunate outcome. This case highlights the complexities in managing recurrent metastatic ES, emphasizing the need for tailored multidisciplinary approaches and early detection strategies.

## Introduction

Ewing's sarcoma, the second most frequent malignant bone tumor following osteosarcoma, is a type of primitive neuroectodermal cancer originating in soft tissues. It primarily impacts children and adolescents. With 95% of the population under 25, it is characterized by high rates of development and growth [1-8]. Males are 1.5 times more inclined than females to develop it [1,5,8]. ES comprises 10-15% of all bone sarcomas. Additionally, it frequently affects the femur, pelvis, humerus, tibia, and fibula [1,2,9,10]. It is primarily seen in the long bones of the extremities. While the ilium and the diaphysis of the femur and tibia are among the most frequently impacted skeleton components, they may impact any portion of the body [3,8].

The European Intergroup Cooperative Ewing Sarcoma Studies (EI-CESS) found that 54% of ES cases occurred in the axial skeleton, 42% in the appendicular skeleton, and the rest in other bones [2,7,9]. Furthermore, only 3.5% to 14.9% of all primary bone sarcomas are primary malignant sarcomas of the spine [2-4]. The two categories of ES of the spine are sacral (sacral and coccygeal) and non-sacral (cervical, thoracic, and lumbar). Additionally, ES of the sacral spine, which is exceedingly aggressive, has a bad prognosis and is less sensitive to treatment. It is more common than ES of the non-sacral spine, which is incredibly uncommon and makes up only 0.9% of all instances [3,5,7,11].

Patients often report swelling or soreness of the affected extremities, that may worsen over a few weeks to months, and frequently get exacerbated by exertion. These symptoms are frequently intermittent and may be more dire at night [2,10]. Unfortunately, some of these cancers are only discovered after swelling starts to appear, even though they have undergone mild trauma. These swollen areas could be painful and erythematous, and some individuals might notice lymphadenopathy [2,8]. Additionally, aside from the signs and symptoms of a neurological deficit caused by spinal cord compression, individuals with lesions involving the spine or sacrum may also experience radiculopathy or back pain. In young patients, this delay in onset has been more common than an acute, rapid progression of the lesion [2,7,10]. The Ewing group of cancers has a defective ES gene (EWSR1) allele. The EWSR1 gene, located on chromosome 22q12, was translocated in the majority of cases. To navigate diagnosis and management, classifications of bone and soft tissue tumors based on these genetic anomalies have significant potential [2].

Radiation, surgery, and chemotherapy are aggressively used as part of the ES treatment plan, which gives patients devoid of metastases a 50%-60% chance of long-term, relapse-free survival. To optimize patient outcomes, the lesion must be found as soon as possible during imaging [8]. Although it is generally agreed that chemotherapy should be used as the first line of treatment since it has been demonstrated to increase survival rates upto 70% [4,12], there is still disagreement over the use of radiation therapy and surgery as follow-up treatments and how much each procedure ought to cost [4]. We present a case of an 18-year-old male with recurrent metastatic ES who presented with fever, cough, and a right humerus lesion. Computed tomography (CT)-guided biopsies confirmed ES presence. Despite prior treatments and complications including spinal metastasis and cord compression, the patient's condition deteriorated, leading to his unfortunate death.

## Case presentation

We present a case of an 18-year-old male with recurrent diagnosed metastatic ES, who was admitted with fever and cough. He complained of non-productive cough, fatigue, mild nasal congestion, shortness of breath, occasional headaches, decreased appetite, and back pain that was initially thought to be a pulled muscle. He had experienced a fever, reaching a maximum temperature of 103.8°F, which responded well to acetaminophen. He also reported nausea and occasional vomiting, although these were considered baseline symptoms.

**Figure 1:**
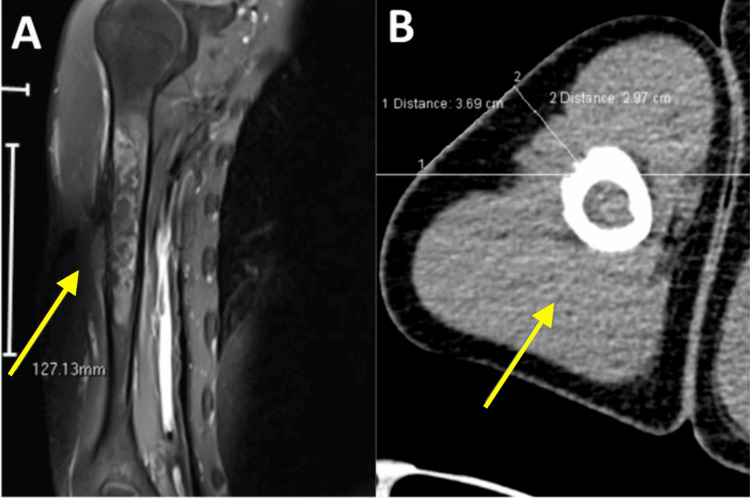
Magnetic resonance imaging (MRI) scan revealed a lytic necrotic lesion characterized by hyperintense signal intensity on T2-weighted images in the right humerus (arrow). Sagittal (A) and axial views ( B).

His past diagnosis of metastatic ES was confirmed through the diagnostic evaluation of a right humerus lesion on a magnetic resonance imaging (MRI) scan, which unveiled significant findings as shown in Figure 1. A CT-guided fine-needle aspiration (FNA) and a core needle biopsy performed at the same site exhibited cellular features characteristic of ES, including clusters of monomorphic cells with round irregular nuclei, fine chromatin, and limited cytoplasm. Notably, approximately 60% of the core biopsy tissue comprised viable tumor cells. These histological findings strongly corroborate the patient's existing diagnosis of ES, confirming its presence in the right humerus region. This aggressive malignancy primarily impacts bone and soft tissues in addition to several other organs.

**Figure 2:**
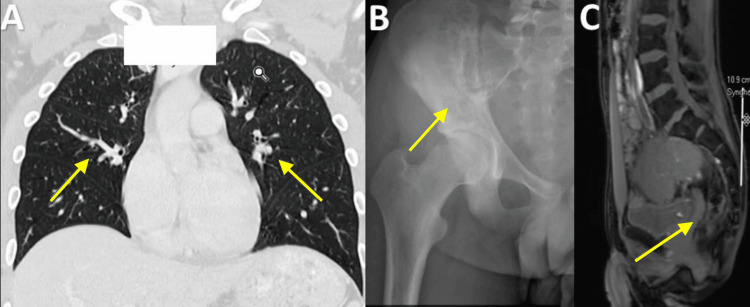
Computed tomography (CT) scan showing lung metastasis (A) and x-ray (B) and CT scan showing bone metastasis in the ischial tuberosity from ES (C).

**Figure 3:**
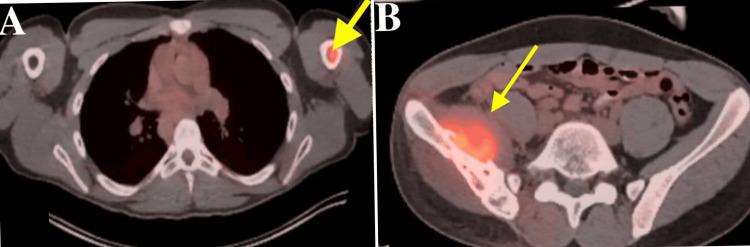
Positron Emission Tomography (PET) scan (A) pinpoints the presence of an aggressive ES lesion within the right humerus, illuminated by hypermetabolic activity, and (B) reveals additional areas of concern with metastasis to ischial tuberosity.

He had a prior history of lung metastasis and bone metastasis to the ischial tuberosity (Figure 2,3). He had received radiation therapy for the metastatic sites to the lungs and iliac region in the past.

**Figure 4:**
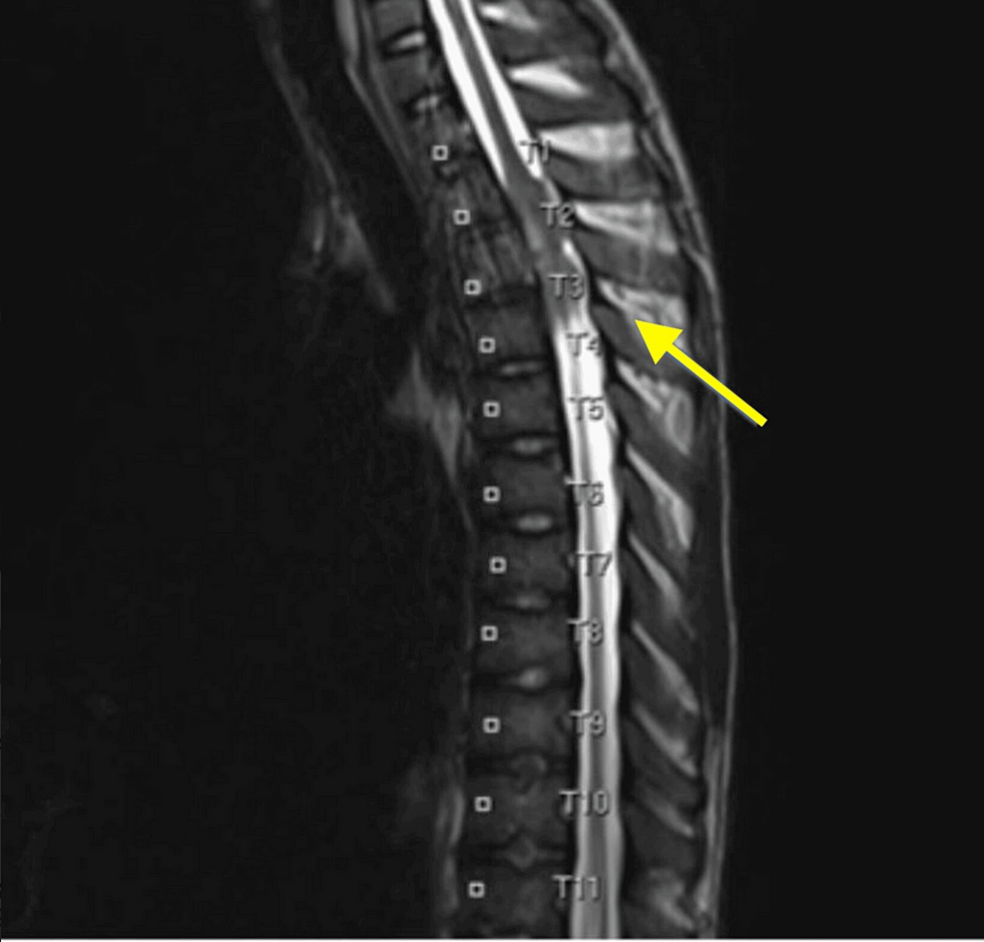
Magnetic resonance imaging (MRI) scan showing metastatic spinal cord compression.

The patient was treated with whole lung radiation and proton therapy to the right ilium delivering a total dose of 4760 cGy to 6020 cGy in 28 fractions to the right iliac region, and has experienced a rapid deterioration in their condition. They now face two critical spinal lesions: cauda equina invasion at the L5 to S3 spine and cord compression at the upper thoracic spine (T2 to T3) as shown in Figure 4. Despite high-dose Decadron treatment, their ambulatory status has declined, making surgical intervention unfeasible. Immediate actions include a CT simulation to assess the sacrum and potentially the thoracic spine, efforts to retrieve Digital Imaging and Communications in Medicine (DICOM) data for treatment planning, and the development of an intensity-modulated radiation therapy (IMRT) plan for both lesions, with caution regarding cauda equina dose tolerance. This complex medical situation necessitates urgent multidisciplinary care coordination to address the spinal lesions and optimize treatment. He underwent multiple rounds of radiation therapy. Notably, he received radiation treatment for cord compression involving L5-S2 and T1-T4 spine, as well as palliative radiation to the right upper extremity.

**Figure 5:**
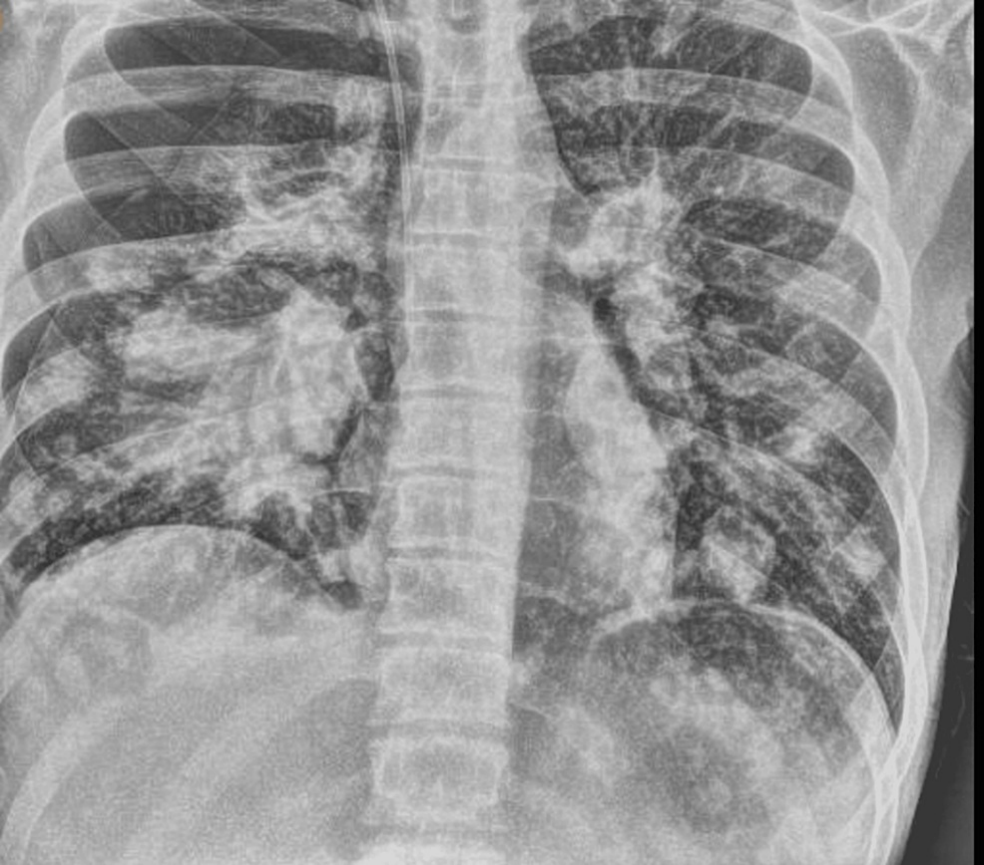
Chest X-ray showed pneumonitis most likely due to radiation.

During his hospitalization, he underwent a bronchoscopy due to concern for pneumonia or the possibility of radiation pneumonitis as shown in Figure 5. His condition improved with antibiotics, and he regained some neurological function in his lower extremities. He was started on high-dose steroids, analgesics, and other supportive medications. Unfortunately, he experienced a fall and developed decreased sensation and motor function in his lower extremities. MRI showed rapid disease progression and spinal cord compression. He received emergent palliative radiation to address the spinal cord compression. There was a plan for further palliative radiation to his right upper extremity for pain relief. However, due to rapid spinal cord compression from the malignant neoplasm metastasizing to the spine and along with hypoxia, the patient succumbed to death despite resuscitative efforts.

## Discussion

ES is the second most common bone neoplasm in the pediatric population, with the highest incidence occurring in the second decade of life, as seen in our patient, and typically involving the long bones of the extremities and pelvis. ES of long bones is generally associated with the expansive nature of the lesion, local swelling, and pain. Loss of range of motion in joints can be caused by juxta-articular lesions, while those involving the ribcage may have extraosseous masses or pleural extension [2,3,9]. Patients with ES of the spine mostly presented with low back pain followed by palpable swelling [2,3]. The primitive neuroectodermal tumor (PNET) type of ES is characterized as a malignant mesenchymal tumor and a member of the "small round-cell tumor" family. The molecular profile of ES is distinct from that of other sarcomas due to the specific translocation featuring FLI1 on chromosome 11q24 and breakpoint region 1 (EWSR1) on chromosome 22. This translocation, known as t(11; 22)(q24; q12), is responsible for 80-90% of cases in which the EWSR1-FLI1 fusion transcript protein is produced, which differentiates ES from other sarcomas and is responsible for its clinical aggressiveness and distinct molecular profile, while the translocation encompassing t(21:22)(q22:q12) fuses EWSR1 and ERG, which is another DNA-binding protein, creating an oncogenic transcription factor that inhibits apoptosis is rare, is associated with 10-20% of cases. There has been a lot of debate about its pathogenesis. James Ewing first suggested an endothelial origin in 1921. Subsequently, the finding of the t(11; 22) translocation led to widespread suspicions of a neural origin. Finally, an epithelial origin was inferred from the erratic expression of cytokeratin and tight junction proteins [3,4,7-13]. Additionally, spinal cord compression-related neurological impairments may cause patients to present later than expected. In our patient's case, worsening bilateral lower limb weakness was noticed, which was preceded by general symptoms like a non-productive cough, fatigue, mild nasal congestion, shortness of breath, sporadic headaches, nausea and sporadic vomiting, reduced appetite, back pain, and fever, which peaked at 103.8°F.

On light microscopy, small, poorly differentiated round cells that form nests, sheets, lobules, or sporadic rosettes are evident. These cells have a high nucleus-to-cytoplasm (N/C) [1] ratio, mitotic figures, and sparse cytoplasm. Scattered cytoplasmic organelles are visible under electron microscopy, along with growth cones that may be signs of differentiation of glial cells [11]. For the identification of destructive lesions with weak circumscription that have been described as having a "moth-eaten" appearance, a plain radiograph should be done first. In addition to an "onion-peel" appearance brought on by periosteal response, ES is also known to have a codman's triangle appearance, which typically denotes an aggressive bone lesion. In most cases, a CT scan is required to ensure a better delineation of the actual damage to the cortices, while an MRI is preferable for detecting soft tissue involvement and disease staging because it provides more precise information on tumor sizes and extension. Further, MRI can aid in planning biopsies and surgical resection by establishing the connection between a lesion and nearby neurovascular systems. Moreover, CT scans can show lung involvement, metastases, bone erosions, and intra-tumoral calcification. Nonetheless, pathological fractures are known to occur in 10% to 15% of individuals after diagnosis [2,8].

The tumor expresses CD99/MIC2 90% of the time. Other positive indicators include the neuronal markers S-100 and synaptophysin, as well as vimentin. Additionally, non-specific CD-99 staining can be seen in alveolar rhabdomyosarcoma, undifferentiated carcinoma, and some non-Hodgkin's malignant lymphomas [3,7,8,11]. These diagnoses were ruled out by the absence of the necessary markers, such as CD45, desmin, and epithelial membrane antigen (EMA) [2], as well as by negative synaptophysin. Additionally, cytogenetic investigation using the fluorescence in situ hybridization (FISH) method helps confirm the diagnosis of ES. The reciprocal translocation t(11; 22)(q24; q12) involving chromosome 22 located on EWS-FLI1 may be found using this method 80-90% of the time [3]. Other conditions similarly associated with ES include giant cell tumor, aneurysmal bone cysts, Langerhans cell histiocytosis, vertebral hemangioma, chordoma, metastatic embryonal rhabdomyosarcoma, neuroblastoma, and bacterial infection.

The treatment of ES must be tailored to the patient's age, location, and size of the tumor, neurological abnormalities, possibilities for reconstruction, the surgeon's experience, clinical history, tumor extension, and the viability of total tumor removal [4,6]. Furthermore, due to their invading behavior as well as their intimacy with essential spinal cord and nerve root structures, ES, like osteogenic sarcoma (OGS) of the osseous spine, are difficult to deal with and avoid extensively negative surgical margins in a considerable portion of patients. In inoperable cases with severe cord compression, radiotherapy is probably utilized more frequently for locoregional management [4]. Due to the challenging nature of the resection, surgical interventions are not frequently provided to patients with spinal involvement. It could be difficult to achieve negative margins without sacrificing functionality. Nevertheless, in some circumstances, palliative stabilization of the spine for the preservation of the spinal cord and other structures is necessary, combined with an adequate distribution of stress across the vertebral column. The degree of spinal stability will be determined by the spinal instability neoplastic scoring (SINS), which consists of six criteria with separate scores. SINS can potentially be used to assess the necessity of spinal stabilization. The score goes from 0 to 18, with a score of 6 or less being considered stable, a score of 7 to 12 denoting unsure or uncertain stability, and a score of 13 to 18 denoting an unstable spine. A patient who receives a score of 8 is frequently strongly advised to have surgery to stabilize their spine. Modern surgical technology and techniques have made it possible to perform treatments quickly and with few side effects [2]. The existence of neurological impairments, which, once apparent, are frequently rapidly progressing, is the deciding factor in the therapy of Ewing's sarcoma of the flexible spine. Only quick surgical decompression can provide the best chance of restoration in such cases. Further, the type of injury determines the strategy of management. When cord compression is brought on by body extension, anterior decompression is necessary. It is noteworthy that ES frequently tends to enter the spinal canal through the intervertebral foramen, compressing the cord radially when it does so. In such cases, laminectomy offers a successful method for cord decompression [2,3].

Neoadjuvant chemotherapy has a better overall survival rate than primary surgery. Europeans advocate the use of vincristine, ifosfamide, doxorubicin, and etoposide (VIDE), while Americans have been known to administer neoadjuvant and adjuvant chemotherapy using vincristine, doxorubicin, and cyclophosphamide (VDC), with alternating cycles of ifosfamide and etoposide (IE) [2,3]. According to the IESS-III research, IE in addition to VDCA (vincristine, doxorubicin, cyclophosphamide, and dactinomycin) has a better five-year relapse-free survival rate than the group receiving only VDCA [2]. A higher rate of en bloc resections with an R0 margin, a separate prognostic indicator for better overall survival, is also a result of induction chemotherapy. Surgery, radiation, or both may be performed after this based on the patient's history. Due to the possibility of post-treatment edema causing neurological compression to establish or develop, primary radiation is not advised in certain circumstances [3].

Due to late presentation and diagnosis, it has also been reported that many patients have already advanced into the metastatic stage of their cancer. Relapse rates for individuals receiving local therapy alone range from 80% to 90%. Hence, multimodal treatment is typically advised [2]. While tumor size in ES exhibited no predictive potential for survival across both OS and DSS, age at diagnosis, surgical resection, and the extent of duration were discovered to be statistically and clinically important survival predictors for ES of the spine [4]. The discovery of necrotic tissue in surgically eliminated specimens and the improvement of hematological markers (lactate dehydrogenase) and radiographic imaging are indicators of therapy effectiveness. Despite having the ability to decrease or control tumor size, radiation must be used with caution when addressing ES to prevent further injury to the patient [2]. Furthermore, radiation exposure and the possibility of additional cancers ought to be a concern for post-treatment surveillance, particularly in younger patients [2]. Late recurrence (>2 years from diagnosis) is associated with longer survival than early recurrence. Similarly, those with elevated LDH at diagnosis and those who underwent en bloc spondylectomy rather than decompression or laminectomy had lower recurrence rates. Therefore, multiple factors should be considered for better oncological control and preservation of spine biomechanics. Further prospective studies should be sought to generate evidence-based treatment guidelines and determine the long-term treatment effects of those modalities [7,12].

## Conclusions

In summary, ES poses a significant challenge, primarily affecting young individuals, and is characterized by its aggressive nature and propensity for metastasis. This case report highlights the critical importance of early detection and proactive treatment strategies in managing recurrent metastatic ES, particularly when it involves the spine. The distinct molecular marker, the EWSR1-FLI1 fusion transcript protein, plays a pivotal role in diagnosis. Timely identification is crucial to prevent metastatic progression and improve outcomes. Treatment necessitates a multifaceted approach, combining neoadjuvant chemotherapy, surgery, and radiation therapy, with treatment decisions influenced by various factors. Balancing oncological control with spinal biomechanics preservation is a delicate task, especially in spinal ES cases. Recent advances, such as craniospinal axis radiotherapy, offer hope for enhanced survival rates and reduced recurrence, but further research is essential to establish evidence-based guidelines. This case emphasized the urgency of early detection, comprehensive care, and ongoing research efforts in tackling ES, with the potential to improve patient outcomes and reduce the burden of recurrent disease.

## References

[1] Zhao DY, Zhang WL, Cui Y, Ma CB, Ni SH, Zhang ZY (2019). Primary Ewing's sarcoma of the vertebral body: A case report. Medicine (Baltimore).

[2] Goh TC, Bajuri MY, Yusof MF, Mohd Apandi H, Sarifulnizam FA (2021). Ewing's sarcoma of the vertebral body in an adolescent: A rare case report and literature review. Cureus.

[3] Cherraqi A, Lemrabet A, Dokal ID, Lrhorfi N, Belghiti H, Allali N, Chat L (2022). Primary Ewing's sarcoma of the spine: About a case. Glob Pediatr Health.

[4] Arshi A, Sharim J, Park DY, Park HY, Yazdanshenas H, Bernthal NM, Shamie AN (2017). Prognostic determinants and treatment outcomes analysis of osteosarcoma and Ewing sarcoma of the spine. Spine J.

[5] Khatavi A, Dhillon CS, Chhasatia N, Pophale C, Nanakkal S, Varshney A (2020). Primary Ewing’s sarcoma of the C2 vertebra with progressive quadriparesis: Report of a rare case and review of the literature. Surg Neurol Int.

[6] Eloqayli H (2017). Adult primary cervical extra-osseous Ewing's sarcoma: A case report and short literature review. Int J Surg Case Rep.

[7] Mandal S, Baniya S, Rohita DK, Yadav GK, Lowry P (2022). A case report on non-metastatic Ewing sarcoma of the lumbar spine in a young patient. Cancer Rep (Hoboken).

[8] Muhuesein TM, Ilangovan G, Arul Pitchai AD, Parthasarathy EA, Anand R, Khalil-Khan A (2022). Extraskeletal Ewing's sarcoma with vertebral metastasis: A case report. Cureus.

[9] Lath N, Joshi PR, Kumari K, Neupane S, Ranjan R, Ghimire N (2023). Thoracic epidural Ewing sarcoma mimicking an epidural abscess: A case report. Int J Surg Case Rep.

[10] Oliveira C, Vital L, Serdoura F, Pinho AR, Veludo V (2020). Spondylectomy for primary Ewing lumbar sarcoma in children. Rev Bras Ortop (Sao Paulo).

[11] Farooq M, Mustafa B, Sultan KA, Ashraf M, Ashraf N, Siddique A (2021). Extraosseous extradural Ewing sarcoma of the thoracic spine: Case report and literature review. Surg Neurol Int.

[12] Jain S, Kapoor G (2010). Chemotherapy in Ewing's sarcoma. Indian J Orthop.

[13] Carballo Cuello CM, De Jesus O, de Jesús Espinosa A, Fernández-de Thomas RJ, Murray G, Pastrana EA (2022). Prognosis and outcome of cervical primary extraosseous intradural extramedullary Ewing sarcoma: A systematic review. Cureus.

